# Pylorus-Preserving Pancreatoduodenectomy with Arterial Reconstruction and Right Gastric Vessel Preservation for Pancreatic Head Cancer with Atherosclerotic Celiac Axis Stenosis and Common Hepatic Artery Occlusion: A Case Report

**DOI:** 10.70352/scrj.cr.26-0222

**Published:** 2026-07-17

**Authors:** Hiroki Nakahara, Takayuki Minami, Naohiro Akita, Hiroaki Usui, Mizuo Hashimoto, Tetsushi Mizutani

**Affiliations:** 1 Department of Surgery, Chubu Rosai Hospital, Nagoya, Aichi, Japan; 2 Division of Vascular and Endovascular Surgery, Department of Surgery, Nagoya University Graduate School of Medicine, Nagoya, Aichi, Japan

**Keywords:** pancreatic head cancer, pancreatoduodenectomy, celiac axis stenosis, common hepatic artery occlusion, arterial reconstruction, gastroduodenal artery stump, right gastric artery preservation

## Abstract

**INTRODUCTION:**

Pancreatoduodenectomy in patients with celiac axis (CA) stenosis or occlusion carries a risk of ischemic complications because hepatic arterial flow may depend on collateral circulation from the superior mesenteric artery. Although securing hepatic arterial flow is essential, preservation of gastric perfusion has rarely been emphasized. Herein, we report a case of pancreatic head cancer with severe atherosclerotic CA stenosis and common hepatic artery (CHA) occlusion, in which arterial reconstruction (ARc) and preserving the right gastric vessels were combined to maintain both hepatic and gastric perfusion.

**CASE PRESENTATION:**

A 76-year-old man was referred to our hospital after an incidental pancreatic head mass was detected using CT. Dynamic contrast-enhanced CT revealed a 15-mm hypovascular tumor in the pancreatic head with dilation of the main pancreatic duct and no major vascular invasion or distant metastasis. Preoperative imaging revealed severe atherosclerotic CA stenosis and CHA occlusion, with marked development of the pancreatoduodenal arcade, suggesting dependence on collateral flow. The right gastric artery (RGA) was relatively large. A pylorus-preserving pancreatoduodenectomy (PPPD) was performed. The ARc was established between the right common iliac artery (RCIA) and the gastroduodenal artery (GDA) stump using a great saphenous vein graft. The RGA and vein were preserved to maintain gastric perfusion. Intraoperative Doppler ultrasonography and indocyanine green fluorescence imaging confirmed adequate hepatic and gastric perfusion, respectively. The operation time was 582 min, with blood loss of 252 mL. Histopathological examination revealed a moderately differentiated adenocarcinoma with lymph node metastasis (pT1N1M0, Stage IIB). All resection margins were pathologically negative, and an R0 resection was achieved. The postoperative course was uneventful, and postoperative CT demonstrated adequate organ perfusion and bypass graft patency. The patient remained recurrence-free for 5 months after surgery.

**CONCLUSIONS:**

PPPD with an ARc between the RCIA and GDA stump, combined with right gastric vessel preservation, enabled safe resection in a patient with severe CA stenosis and CHA occlusion. Preoperative hemodynamic assessment may help guide individualized revascularization and vessel preservation. However, because direct intraoperative assessment of gastric perfusion under RGA clamping was not performed, the necessity of RGA preservation could not be objectively confirmed in this case.

## Abbreviations

ALT
: alanine aminotransferase
ARc
: arterial reconstruction
AST
: aspartate aminotransferase
CA
: celiac axis
CHA
: common hepatic artery
DGE
: delayed gastric emptying
DP-CAR
: distal pancreatectomy with celiac axis resection
GDA
: gastroduodenal artery
GSV
: great saphenous vein
ICG
: indocyanine green
IPDA
: inferior pancreaticoduodenal artery
LGA
: left gastric artery
PD
: pancreatoduodenectomy
PPPD
: pylorus-preserving pancreatoduodenectomy
RCIA
: right common iliac artery
RGA
: right gastric artery
RGEA
: right gastroepiploic artery
SA
: splenic artery
SMA
: superior mesenteric artery
SMV
: superior mesenteric vein
UICC
: Union for International Cancer Control

## INTRODUCTION

CA or SMA stenosis has been reported in 11% of patients undergoing PD,^1)^ and its prevalence is presumed to be even higher in aging societies. When PD is performed in patients with hemodynamically severe CA stenosis, securing hepatic arterial flow is crucial for preventing ischemic complications. Herein, we report a case of PPPD for pancreatic head cancer with severe atherosclerotic CA stenosis and CHA occlusion, in which an ARc was performed between the RCIA and the GDA stump, along with preservation of the right gastric vessels to maintain gastric perfusion.

## CASE PRESENTATION

A 76-year-old man was referred to our department after a pancreatic head mass was incidentally detected on a CT performed during the evaluation for prostate cancer. Dynamic contrast-enhanced CT revealed an irregular hypovascular tumor measuring 15 mm in the pancreatic head with dilatation of the main pancreatic duct (**Fig. 1A**). The tumor did not involve major vessels, including the GDA, CHA, CA, SMA, SMV, or portal vein. No biliary dilatation was observed. There was no evidence of distant metastases, such as liver or lung metastases. Endoscopic ultrasonography revealed a heterogeneous, hypoechoic mass in the pancreatic head. Pancreatic juice and brush cytology of the pancreatic duct stricture revealed atypical cells with nuclear enlargement and loss of polarity. Blood examinations showed that carcinoembryonic antigen, carbohydrate antigen 19-9, DUPAN-2, and Span-1 levels were within normal ranges. No elevation in hepatobiliary enzyme levels was observed. Based on these findings, the patient was diagnosed with resectable pancreatic head cancer (cT1cN0M0, cStage IA) according to the criteria of the UICC, 8th edition. The patient had a history of malignant lymphoma that was treated 30 years previously and was currently in remission. As standard preoperative chemotherapy for pancreatic cancer, gemcitabine plus S-1 was initiated (gemcitabine 1000 mg/m^2^ on days 1 and 8 and S-1 100 mg/body daily on days 1–14 of a 21-day cycle). However, the regimen was discontinued after 1 course because grade 3 neutropenia occurred. CT after preoperative chemotherapy demonstrated no substantial change in tumor size, with no newly developed vascular involvement or distant metastasis; therefore, the tumor remained resectable. Preoperative CT images also revealed severe CA stenosis due to atherosclerotic changes, and the CHA was occluded (**Figs. 1B**, **1C**, **2A**, and **2B**). These findings suggest that chronic hypoperfusion of the CA system may lead to CHA atrophy and subsequent occlusion. The pancreaticoduodenal arcade was markedly developed (**Figs. 1D**, **1E**, and **2C**). The RGA was relatively enlarged (**Fig. 2B**). The pancreatic tumor was in contact with the root of the common trunk of the IPDA and the first jejunal artery, precluding the preservation of the pancreatic head arcade (**Fig. 2A**). The dorsal pancreatic artery arising from the SMA was not well developed. Because hepatic arterial flow was expected to decrease after division of the GDA during PD, an ARc was planned between the RCIA and the GDA stump using a GSV graft. Additionally, considering the risk of gastric ischemia postoperatively if the RGA was divided, preserving the RGA and vein to maintain gastric perfusion through the arterial arcade was selected. After multidisciplinary discussion with vascular surgeons, this strategy was favored over attempting to improve LGA blood flow by CA stenting.

**Fig. 1:**
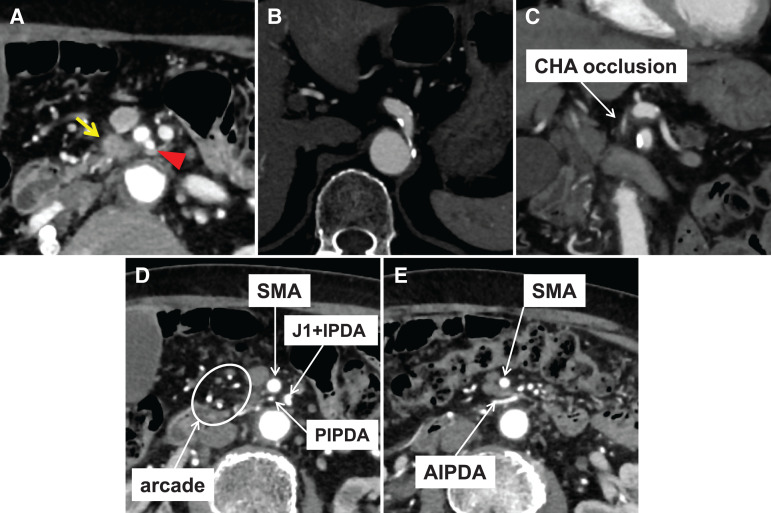
Preoperative CT images show (**A**) an irregular nodular tumor (arrow) of 15 mm in the pancreatic head in contact with the root of the common trunk of the IPDA and the first jejunal artery (arrowhead), (**B**) the CA stenosis due to atherosclerosis, (**C**) CHA atrophy and occlusion, and (**D**, **E**) developed pancreaticoduodenal arcade. AIPDA, anterior inferior pancreaticoduodenal artery; CA, celiac axis; CHA, common hepatic artery; IPDA, inferior pancreaticoduodenal artery; J1, first jejunal artery; PIPDA, posterior inferior pancreaticoduodenal; SMA, superior mesenteric artery

**Fig. 2:**
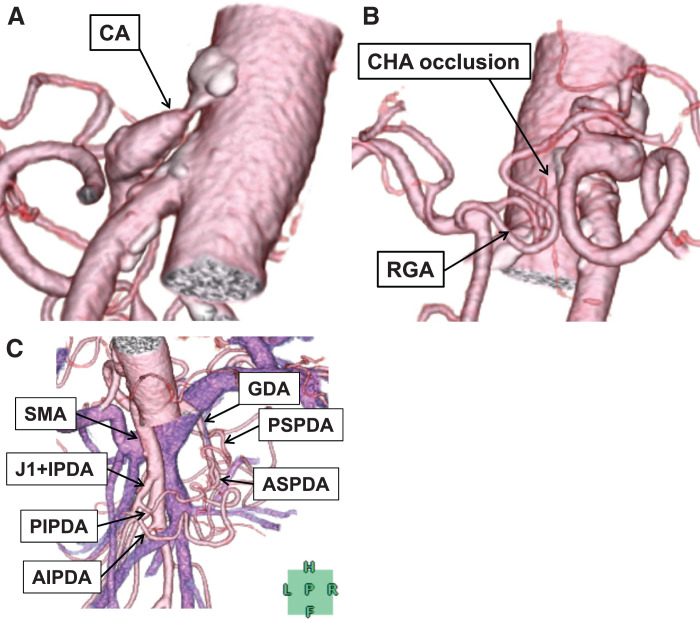
3D reconstruction imaging. (**A**) Severe stenosis with post-stenotic dilatation is observed at the CA. (**B**) The CHA is atrophic and occluded. The RGA is relatively enlarged, and a well-developed collateral arcade between the left and right gastric arteries is observed. (**C**) The pancreaticoduodenal arcade appears markedly developed. AIPDA, anterior inferior pancreaticoduodenal artery; CA, celiac axis; CHA, common hepatic artery; GDA, gastroduodenal artery; IPDA, inferior pancreaticoduodenal artery; PIPDA, posterior inferior pancreaticoduodenal artery; RGA, right gastric artery; SMA, superior mesenteric artery

A midline upper abdominal incision was made. Intra-abdominal exploration revealed no liver metastases or peritoneal dissemination. Peritoneal washing cytology results were negative. PPPD was performed while preserving the RGA and vein. The left gastric vein was preserved. During surgery, test clamping of the GDA resulted in decreased hepatic arterial flow, confirmed using intraoperative pulsed Doppler ultrasonography, as well as reduced blood flow in the RGA. Therefore, the ARc was performed as planned. The reconstruction procedures were performed after specimen removal. To minimize the ischemic time in both the liver and stomach, the division of the GDA and IPDA was postponed until the late phase of the procedure. In parallel, the left GSV graft was harvested simultaneously by a vascular surgeon. After transecting the pancreas just above the portal vein, the GDA and IPDA were divided, the pancreatic head nerve plexus was dissected, and the specimen was removed (**Fig. 3A**). For the ARc, approximately 15 mm of the GDA stump was preserved. Subsequently, the ARc was performed. Unfractionated heparin (total dose, 4000 units) was intravenously administered as a bolus immediately before the ARc, and the activated coagulation time was prolonged to 216 s. The graft was anastomosed to the RCIA using continuous 6-0 Prolene sutures, followed by end-to-end anastomosis between the graft and the GDA stump using continuous 7-0 Prolene sutures. The graft was routed through the retroperitoneal space adjacent to the inferior vena cava. A vascular surgeon performed ARc using surgical loupes (**Fig. 3B** and **3C**). After the reconstruction, adequate hepatic arterial flow was confirmed using pulsed Doppler ultrasonography. ICG fluorescence imaging demonstrated good visualization of the graft and remnant stomach, indicating preserved blood flow to the remnant stomach (**Fig. 4A** and **4B**). ICG fluorescence assessment was not performed during temporary clamping of the RGA; therefore, gastric perfusion after interruption of RGA flow could not be directly evaluated intraoperatively. After ARc, gastrointestinal reconstruction was performed using the modified Child’s method. Following the hepaticojejunostomy, a pancreatojejunostomy was performed using a pancreatic duct-to-jejunal mucosa anastomosis with the modified Blumgart method. The lost stent (7.5 Fr) was placed through the main pancreatic duct into the intestinal tract. The pancreatic parenchyma was firm and fibrotic. Closed-suction silicone drains (6.5 mm in diameter) were placed anterior and posterior to the pancreatojejunostomy and at the foramen of Winslow. In addition, the round ligament was interposed between the GDA stump–GSV graft anastomosis and the pancreatojejunostomy to separate these structures (**Fig. 5**). In conventional PPPD, the remnant stomach typically assumes a vertically oriented configuration along the cranio-caudal axis, making the duodenal stump the most caudal portion of the remnant stomach; therefore, a duodenojejunostomy is usually performed. However, preservation of the RGA altered the configuration of the remnant stomach, resulting in a more horizontally oriented axis. Consequently, the lower gastric body became the most caudal portion of the remnant stomach, and gastrojejunostomy was performed at this site with a side-to-side anastomosis (**Fig. 6**). The operation time was 582 min, and the blood loss was 252 mL. No blood transfusion was required. No postoperative antithrombotic agents were administered. Histopathological examination revealed a moderately differentiated adenocarcinoma. Despite preservation of the RGA, lymphadenectomy of nodal stations #8a, #8p, #12, #13, #14, and #17 was successfully performed. A total of 12 lymph nodes were retrieved, and metastatic adenocarcinoma was identified in 1 lymph node at station #13. All resection margins were pathologically negative, and an R0 resection was achieved. The final pathological diagnosis was pT1N1M0 (pStage IIB), according to the UICC, 8th edition (**Fig. 7A** and **7B**).

**Fig. 3:**
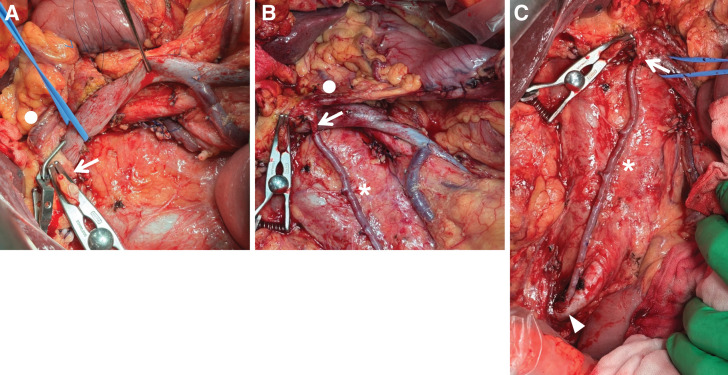
Intraoperative view (**A**) after specimen removal, (**B**, **C**) ARc between the GDA stump and the RCIA using a GSV graft (circle: right gastric vessels; arrow: GDA stump; arrowhead: RCIA; *: GSV graft). ARc, arterial reconstruction; GDA, gastroduodenal artery; GSV, great saphenous vein; RCIA, right common iliac artery

**Fig. 4:**
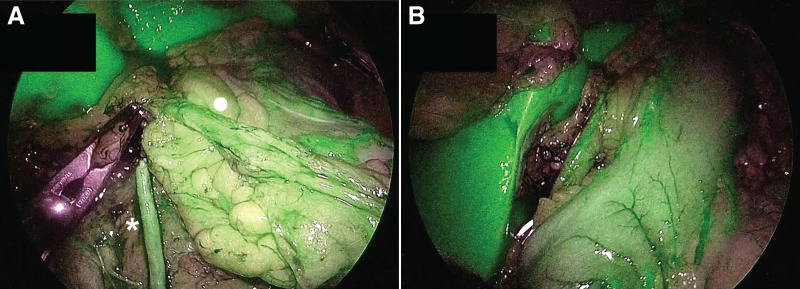
Intraoperative ICG fluorescence imaging. (**A**, **B**) ICG fluorescence signal in the GSV graft (*), RGA (circle), and remnant stomach was sufficient to confirm adequate perfusion. GSV, great saphenous vein; ICG, indocyanine green; RGA, right gastric artery

**Fig. 5:**
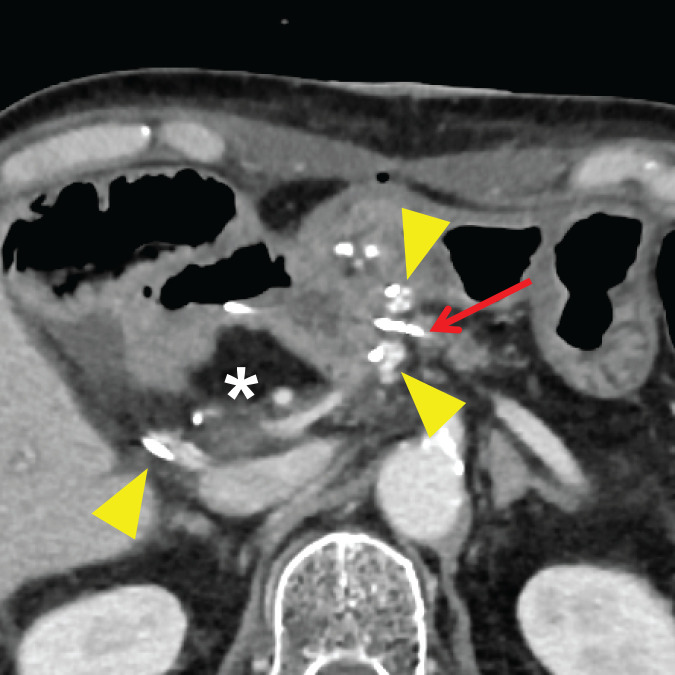
Postoperative axial CT image showing the placement of drains around the PJ and interposition of the round ligament between the PJ and graft anastomosis (arrow: PJ; arrowhead: drains around PJ; *: graft). PJ, pancreatojejunostomy

**Fig. 6:**
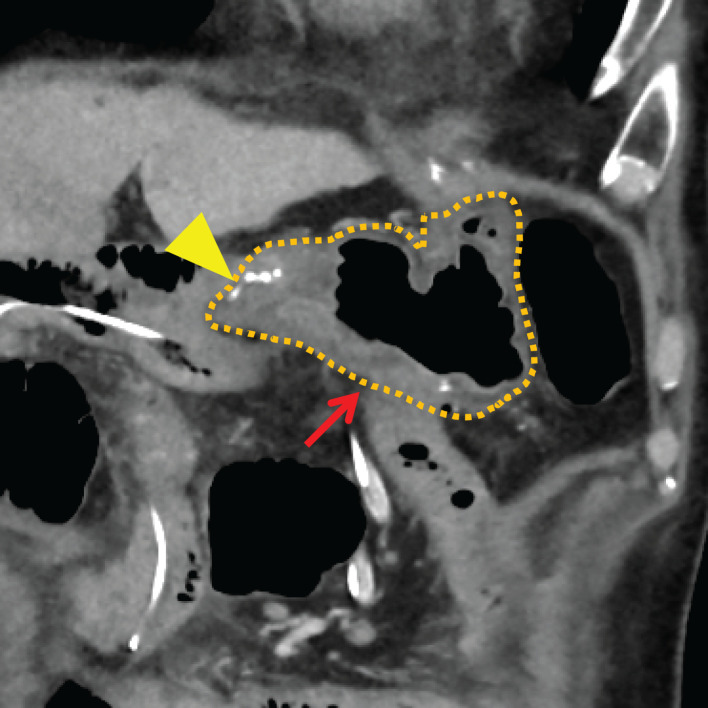
Postoperative coronal CT image showing that the preservation of the RGA altered the remnant stomach from its usual vertical orientation to a more horizontal configuration, resulting in the lower gastric body becoming the most caudal portion, where GJ was performed (arrow: GJ; arrowhead: duodenal stump; dotted line: remnant stomach). GJ, gastrojejunostomy; RGA, right gastric artery

**Fig. 7:**
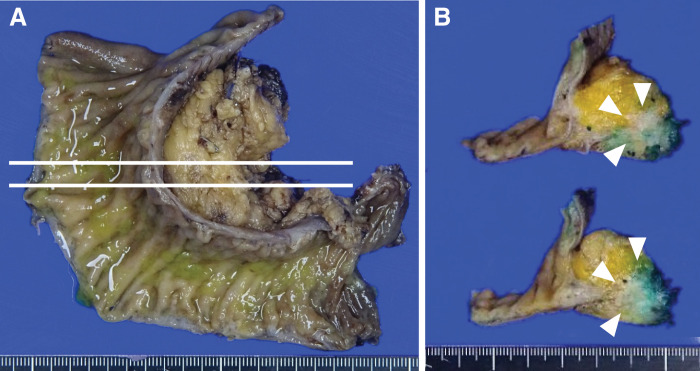
Macroscopic findings of the resected specimen. (**A**, **B**) Macroscopic findings of the resected specimen show an irregular nodular tumor in the pancreatic head (arrowheads). (**B**, cross-section at line)

The postoperative course was uneventful. Serum AST and ALT levels peaked at 433 and 344 U/L, respectively, on POD 2, but promptly returned to normal ranges thereafter. No postoperative liver failure or abscesses were observed. The patient began oral intake on POD 4 without DGE or ischemic gastric complications. Dynamic CT obtained on PODs 3 and 10 confirmed adequate organ perfusion and bypass graft patency (**Fig. 8**). The patient was discharged on POD 17. S-1 adjuvant chemotherapy was initiated 6 weeks postoperatively. Contrast-enhanced CT performed 4 months postoperatively also confirmed the patency of the bypass graft. The patient remained alive without recurrence for 5 months after surgery.

**Fig. 8:**
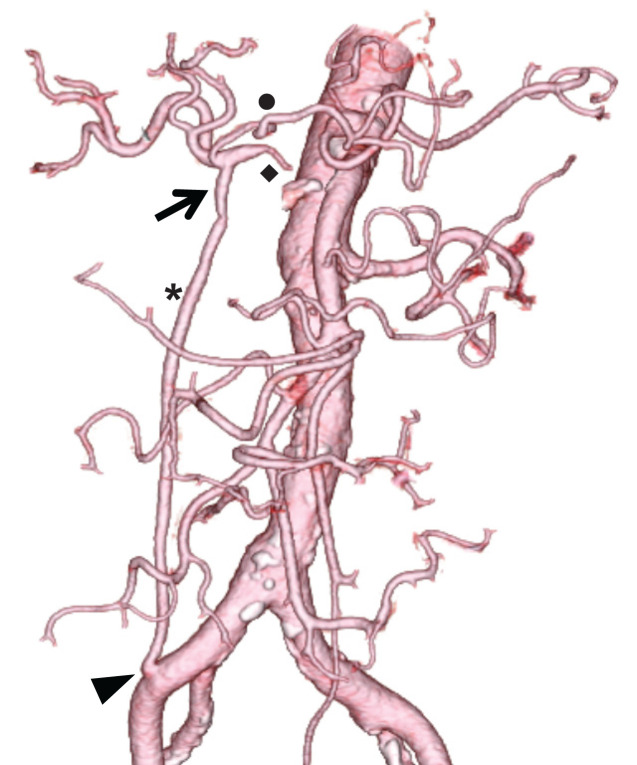
Postoperative 3D reconstruction image. Dynamic CT obtained on PODs 3 and 10 shows adequate organ perfusion and patency of the bypass graft (circle: right gastric vessels; arrow: GDA stump; arrowhead: RCIA; *: GSV graft; ◆: CHA occlusion). CHA, common hepatic artery; GDA, gastroduodenal artery; GSV, great saphenous vein; RCIA, right common iliac artery

## DISCUSSION

This case demonstrates that PD can be safely performed in patients with severe atherosclerotic CA stenosis and CHA occlusion when surgical planning is guided by meticulous preoperative hemodynamic assessment. In the present case, preoperative imaging findings were suggestive of collateral pathways and relative arterial contributions, which enabled the selection of the ARc between the RCIA and GDA stump, together with preservation of the RGA to maintain both hepatic and gastric perfusion.

During PD, the pancreaticoduodenal arcade and GDA are divided. Therefore, in patients with stenosis or occlusion of the CA system, hepatic arterial flow may depend on collateral circulation, placing them at risk of postoperative ischemic complications. CA or SMA stenosis has been reported in a proportion of patients undergoing PD, and the importance of prevention and early management of ischemic complications has been emphasized.^1,2)^ The causes of CA stenosis include extrinsic compression by the median arcuate ligament and atherosclerotic disease.^3)^ Accordingly, revascularization strategies for CA stenosis include division of the median arcuate ligament, endovascular stent placement, and surgical bypass.^4–7)^ In the present case, CA stenting was considered a possible treatment option. Improvement of the LGA flow by CA stenting might have allowed dividing the RGA, thereby reducing oncological concerns related to preserving the RGA. However, after multidisciplinary discussion with vascular surgeons, surgical bypass reconstruction combined with preserving the RGA was selected. Severe CA stenosis often requires technically demanding endovascular procedures through collateral pathways rather than a straightforward antegrade approach.^4,5)^ Furthermore, dual antiplatelet therapy is required after stent placement, and concerns remain regarding long-term patency compared with surgical bypass reconstruction.^8,9)^ Therefore, ARc combined with preserving the RGA was selected as 1 possible strategy for maintaining both hepatic and gastric perfusion in the present case. Several techniques for hepatic arterial revascularization during PD have been reported, including direct anastomosis; arterial transposition using the GDA, RGEA, LGA, SA, middle colic artery, and jejunal artery; and bypass with a vascular graft.^10–12)^ Furthermore, arterial bypass has been performed using an autologous saphenous vein graft from the iliac artery during PD in patients with CA occlusion or severe stenosis. Okamoto et al. reported iliac artery–SA bypass using a saphenous vein graft during PD for CA occlusion, whereas Sasaki et al. described RCIA–CHA bypass during PD for pancreatic head cancer with CA stenosis.^13,14)^ In the present case, bypass reconstruction using a GSV graft was selected because this approach was considered capable of providing sufficient hepatic arterial flow without compromising blood supply to the remnant stomach.^12)^ In contrast to previous reports in which the graft was anastomosed to the SA or CHA, the present case used the GDA stump as the outflow site while simultaneously preserving the RGA to maintain gastric perfusion. Accordingly, ARc was performed using a left GSV graft with the RCIA as the inflow and the GDA stump as the outflow. Reconstruction using SMA branches such as the middle colic artery or jejunal artery has been reported as a useful option for hepatic ARc during PD because it avoids vein graft harvesting and requires only a single anastomosis. However, Kimura et al. reported that reconstruction using the second jejunal artery resulted in extensive hepatic infarction, emphasizing the importance of securing sufficient and stable hepatic arterial inflow.^12)^ In addition, SMA branches are important vessels supplying intestinal perfusion, and anastomotic complications such as stenosis or thrombosis may potentially affect mesenteric circulation. Other reconstructive options were considered; however, based on the present vascular anatomy, severe CA stenosis with CHA occlusion, and the anticipated hemodynamic changes after dividing the pancreaticoduodenal arcade, the RCIA–GDA bypass using a GSV graft was selected in the present case. To preserve the RGA, which arises near the bifurcation of the proper hepatic artery and GDA, the GDA was divided, leaving an approximately 15-mm stump to which the graft was anastomosed. Fortunately, as there was no tumor invasion of the GDA, this surgical approach was feasible and safe.

In the present case, ARc was performed after specimen removal. ARc preceding specimen removal may help reduce hepatic ischemic time^12)^; however, it may interfere with the PD procedure and increase the risk of intraoperative injury to the graft or the anastomotic site. In the present case, where reconstruction using a GDA stump was planned, concerns were raised regarding tension, compression, or injury to the reconstructed graft and anastomosis during dissection of the pancreatic head nerve plexus. Therefore, we prioritized the safe completion of tumor resection and performed ARc thereafter. In addition, the division of the GDA and IPDA was postponed until the late phase of the procedure to preserve hepatic and gastric perfusion as long as possible during PD. Furthermore, harvesting of the left GSV graft was performed simultaneously to shorten the interval before revascularization. As a result, the ARc was safely completed, and the postoperative course was uneventful without ischemic complications.

Alongside securing hepatic arterial flow, preservation of gastric perfusion was an important issue in the present case. Preoperative CT revealed a relatively enlarged RGA, suggesting altered collateral hemodynamics associated with severe atherosclerotic CA stenosis and CHA occlusion. Intraoperatively, test clamping of the GDA resulted not only in decreased pulsation of the proper hepatic artery but also in reduced blood flow of the RGA. These findings were considered to suggest that gastric perfusion through the gastric arterial arcade from the LGA was limited and that blood flow to the RGA was highly dependent on collateral circulation through the GDA arcade. Under such hemodynamic conditions, the division of the RGA may potentially result in insufficient gastric blood flow after surgery, particularly during the resumption of oral intake, leading to ischemic gastropathy or DGE. However, because direct assessment of gastric perfusion under temporary RGA clamping was not performed, this possibility remains speculative. Similar concerns have been raised in DP-CAR, in which the LGA, left gastroepiploic artery, and short gastric arteries are divided, and gastric perfusion depends mainly on the RGA and RGEA. Consequently, postoperative ischemic gastropathy has been reported as a clinically relevant complication, and modified DP-CAR procedures preserving the LGA or additional reconstruction of the LGA have been proposed to prevent gastric ischemia.^15–18)^ Similarly, in PD, reports have described preservation or reconstruction of the RGEA in patients undergoing PD after esophagectomy or proximal gastrectomy, as well as preservation of the RGA in PD for patients with CA occlusion, as in the present case.^19–24)^ Thus, the preservation of gastric blood flow and function remains an important issue in pancreatic resection. Assessment of hemodynamic status based on preoperative imaging and careful consideration of whether adequate gastric perfusion is maintained after resection is crucial for preventing ischemic complications. In the present case, preservation of the RGA was selected based on indirect hemodynamic findings, including relative enlargement of the RGA on preoperative imaging and reduced RGA flow during GDA test clamping. Furthermore, intraoperative ICG fluorescence imaging confirmed adequate perfusion of both the bypass graft and the remnant stomach after reconstruction. However, ICG fluorescence assessment was not performed during temporary clamping of the RGA. Therefore, the presence or absence of gastric ischemia after interruption of RGA flow could not be directly evaluated intraoperatively, and the necessity of the RGA preservation could not be objectively confirmed in the present case.

However, preserving the RGA during PD raises concerns regarding oncological adequacy, particularly concerning lymphadenectomy around the CHA, including nodal stations #8a and #8p. In the present case, lymphadenectomy of nodal stations #8, #12, #13, #14, and #17 was performed despite preservation of the RGA. A total of 12 lymph nodes were retrieved; metastatic adenocarcinoma was identified in 1 lymph node at station #13, and pathological examination confirmed an R0 resection. However, preserving the RGA may have limited the extent of dissection around the CHA, and the oncological adequacy of lymphadenectomy in this region remains unclear. Because no additional intraoperative photographs documenting the dissection around the occluded CHA were available, the adequacy of lymphadenectomy in this region could not be objectively demonstrated. The Japanese Clinical Practice Guidelines for Pancreatic Cancer do not recommend prophylactic extended lymphadenectomy, and the clinical significance of prophylactic regional lymph node dissection remains unclear.^25)^ Imamura et al. and Yamada et al. reported that the prognostic impact of lymph node dissection around the hepatoduodenal ligament and CHA region may be limited in selected patients with pancreatic head cancer.^26,27)^ Furthermore, Nagakawa et al. proposed that “regional lymph node dissection” and “dissection to achieve R0 resection” should be considered distinct concepts.^28)^ In the present case, although pathological examination demonstrated an R0 resection, the adequacy of lymphadenectomy around the CHA could not be fully assessed. Pathological confirmation of an R0 resection alone cannot fully validate the oncological adequacy of lymphadenectomy around the CHA. Therefore, preserving the RGA may be considered an option in selected patients when gastric perfusion is a major concern; however, the oncological implications of this strategy require further investigation.

Furthermore, intraoperative ICG fluorescence imaging confirmed adequate perfusion of both the bypass graft and the remnant stomach.^17,29–31)^

This case report has several important limitations. First, ICG fluorescence imaging was not performed during temporary RGA clamping; therefore, whether division of the RGA would have resulted in clinically relevant gastric ischemia could not be objectively determined. Second, although lymphadenectomy, including stations #8a and #8p, was performed and an R0 resection was achieved, preserving the RGA may have limited the extent of dissection around the CHA. Third, additional intraoperative images demonstrating dissection around the occluded CHA were unavailable. Therefore, the oncological adequacy of lymphadenectomy in this region remains unclear. These limitations should be considered when interpreting the significance of the present case.

## CONCLUSIONS

In conclusion, PPPD with an ARc between the RCIA and GDA stump using a GSV graft, combined with preservation of the RGA, was successfully performed in the present case of severe atherosclerotic CA stenosis and CHA occlusion. Preoperative imaging-based assessment of hemodynamics may provide useful information for individualized planning of revascularization and vessel preservation in selected patients undergoing PD. However, because direct assessment of gastric perfusion under RGA clamping was not performed and the oncological adequacy of lymphadenectomy around the CHA remains unclear, the necessity of preserving the RGA should be interpreted with caution.

## DECLARATIONS

### Availability of data and materials

The data are not available for public access because of patient privacy concerns, but are available from the corresponding author upon reasonable request.
